# Transcription factor ZNF25 is associated with osteoblast differentiation of human skeletal stem cells

**DOI:** 10.1186/s12864-016-3214-0

**Published:** 2016-11-04

**Authors:** Natalie A. Twine, Linda Harkness, Moustapha Kassem, Marc R. Wilkins

**Affiliations:** 1 School of Biotechnology and Biomolecular Sciences, University of New South Wales, Sydney, NSW Australia; 2 Department of Endocrinology and Metabolism, Endocrine Research Laboratory (KMEB), Odense University Hospital, Odense, Denmark; 3 Stem Cell Unit, Department of Anatomy, Faculty of Medicine, King Saud University, Riyadh, Saudi Arabia; 4 Present Address: Pluripotent Stem Cell Group, Australian Institute for Bioengineering and Nanotechnology, University of Queensland, Brisbane, QLD Australia

**Keywords:** Osteogenesis, Mesenchymal stem cells, Osteoblasts, *ZNF25*, Human transcription factors

## Abstract

**Background:**

The differentiation of human bone marrow derived skeletal stem cells (known as human bone marrow stromal or mesenchymal stem cells, hMSCs) into osteoblasts involves the activation of a small number of well-described transcription factors. To identify additional osteoblastic transcription factors, we studied gene expression of hMSCs during ex vivo osteoblast differentiation.

**Results:**

Clustering of gene expression, and literature investigation, revealed three transcription factors of interest – *ZNF25, ZNF608* and *ZBTB38*. siRNA knockdown of *ZNF25* resulted in significant suppression of alkaline phosphatase (ALP) activity. This effect was not present for *ZNF608* and *ZBTB38*. To identify possible target genes of ZNF25, we analyzed gene expression following *ZNF25* siRNA knockdown. This revealed a 23-fold upregulation of matrix metallopeptidase 1 and an 18-fold upregulation of leucine-rich repeat containing G protein-coupled receptor 5 and RAN-binding protein 3-like. We also observed enrichment in extracellular matrix organization, skeletal system development and regulation of ossification in the entire upregulated set of genes. Consistent with its function as a transcription factor during osteoblast differentiation of hMSC, we showed that the ZNF25 protein exhibits nuclear localization and is expressed in osteoblastic and osteocytic cells in vivo. *ZNF25* is conserved in tetrapod vertebrates and contains a KRAB (Krueppel-associated box) transcriptional repressor domain.

**Conclusions:**

This study shows that the uncharacterized transcription factor, *ZNF25*, is associated with differentiation of hMSC to osteoblasts.

## Background

Adult human skeletal stem cells (also known as bone marrow stromal or mesenchymal stem cells, hMSCs) are present in the bone marrow stroma. They are defined by their ability to both self-renew and differentiate into mesoderm-specific lineage cells including osteoblasts, adipocytes and chondrocytes [1, 2]. These two characteristics make hMSCs a valuable resource in the fields of cellular therapeutics and regenerative medicine [3, 4]. The potential clinical use of hMSC therapy has been examined in an increasing number of clinical conditions, including treating children with *osteogenesis imperfecta* [5–7] as well as bone repair of non-healed fractures and large bone defects [4, 8, 9].

Lineage-specific differentiation of hMSCs into osteoblasts (OBs) is dependent on a number of microenvironmental cues [1, 10]. In vitro OB differentiation of hMSCs is induced by a mixture of hormones (e.g. dexamethasone, calcitriol) and chemicals (e.g. organic phosphate donors such as β-glycerophosphate) and the expression of mature OB phenotype takes place through a series of developmental stages: cell expansion and proliferation, cell commitment to OB, and differentiation into pre-osteoblasts followed by maturation of osteoblasts which synthesize the bone matrix and promote mineralization [10, 11]. Phases of OB differentiation and establishment of the osteoblastic phenotype are controlled by a set of transcription factors.

A number of transcription factors (TFs) have been demonstrated to play important roles in OB differentiation and function. Runt domain-containing transcription factor *(RUNX2)* is the major TF in both osteoblast commitment and differentiation [10–12]. Homozygous deletion of this gene in mice resulted in a complete absence of osteoblasts and bone formation [12]. Another TF, *Osterix* (*OSX* or *SP7*), specifically expressed by osteoblasts, is positively regulated by and acts downstream of *RUNX2* [10]. Activating transcription factor 4 *(ATF4)* plays an important role in mature osteoblasts, and it interacts with *RUNX2* to regulate the expression of osteocalcin [10]. Other TFs that have been shown to regulate osteoblast differentiation include: the *AP1* family of proteins; *LEF/TCF* (via Wnt signalling); homeobox proteins *MSX2, HOXA2* and *DLX5*; helix-loop-helix (bHLH) proteins *HES, HEY, TWIST* and *HAND2*; and CCAAT/enhancer-binding proteins (*C/EBPs*) [13]. Although a number of TFs have been identified to be important in osteoblastic differentiation, this is a very small subset of all documented human TFs. Vaquerizas et al. [14] have generated a list of 1391 manually curated, sequence-specific DNA-binding human TFs.

Many reported human transcription factors are uncharacterized in terms of their biological functions [14]. It is plausible that some of the uncharacterized TFs are important regulators of osteoblast differentiation. In this study, we employed genome-wide expression profiling to identify TFs which were differentially expressed between undifferentiated hMSCs and their differentiated osteoblastic cell progeny. By clustering these TFs using self-organizing maps (SOMs), and by literature analysis, we identified three TFs as novel candidates with possible regulatory functions in osteoblast differentiation. We further explored the role of one of these candidates, *ZNF25*. si*ZNF25* knockdown experiments showed regulatory effects on osteoblast differentiation. Microarray analysis of si*ZNF25* deficient osteoblastic cells, identified three highly up-regulated genes*, LGR5, MMP1* and *RANBP3L*, and we propose these as possible targets of *ZNF25*. We also report that ZNF25 has a KRAB domain, a transcriptional repressor, which is conserved in tetrapod vertebrates.

## Methods

### Cell culture

As a model for primary hMSCs, we employed hMSC-TERT cells (subclone hMSC-TERT4). The source and generation of hMSC-TERT cells are described in [15]. These exhibit a stable cellular and molecular phenotype comparable to that of primary hMSCs [16]. hMSC-TERT cells were routinely cultured in standard media (SM) (MEM (Invitrogen) with 10 % v/v FBS (PAA, Pasching, Austria). The generation and characterization of hMSC-TERT cells were as described in detail in [15].

### Osteoblast differentiation

Ex vivo osteoblast differentiation was performed using osteoblast induction medium containing β-glycerophosphate (10 mM; Calbiochem-Merck), L-ascorbic acid-2-phosphate (50 μg/ml; Sigma-Aldrich, Brøndby, Denmark), dexamathasone (10nM; Sigma-Aldrich) and calcitriol (1,25 hydroxy-vitamin D_3;_ 10nM) in standard medium (SM). Media were changed every 3 days until day 15.

### Alkaline phosphatase activity measurements

Alkaline phosphatase (ALP) activity was quantified as previously described [17], using a 1 mg/ml solution of P-nitrophenylphosphate (Sigma-Aldrich, Brøndby, Denmark) in 50 mM NaHCO_3_ with 1 mM MgCl_2_, pH 9.6, at 37 °C for 20 min. Activity was stopped using 3 M NaOH and the absorbance of each reaction (max = 405 nm) was measured using a FLUOstar Omega plate reader (BMG Laboratories, Ramcon A/S, Birkerod, Denmark). ALP activity was normalized to cell number, as determined using a CellTiter-Blue Cell Viability assay, according to manufacturer’s instructions (Promega, Nacka, Sweden).

### Cytochemical staining

Cells undergoing osteogenic differentiation were stained at days 6, 10 and 15 for ALP and days 10 and 15 for alizarin red (AZR) as previously described [18]. Elution of AZR staining was performed using 10 % cetylpyridium for 1 h at room temperature; 25–100 μl was then removed to a 96 well plate and read on a FLUOstar Omega plate reader at 595 nm emission wavelength.

### Immunohistochemical staining

Routine protocols [18] were used to stain for *ZNF25* (Novus Biologics antibody H00219749-B01). Briefly, immunocyto-chemical staining was performed using DAKO PowerVision + HRP according to manufacturer’s instructions. The primary antibody was diluted in ChemMate Antibody diluent (S2022, Dako, Glostrup, Denmark) and processed on an automatic slide processor (Techmate500, Dako, Glostrup, Denmark). DAB was used as the chromogen and the slides were counterstained with haematoxylin. Analysis was carried out on an IX50 Olympus microscope using OlympusDP Software v3.1 (Olympus, Essex, UK) or a Leica DM4500 (Leica, Wetzlar, Germany) using the Surveyor Turboscan Mosaic acquisition imaging analysis system v5.04.01 (Objective Imaging Ltd, Cambridge, UK). To assess localization of the ZNF25 protein, cells undergoing OB induction were passaged and replated 2 days prior to fixation (4 % formalin) in osteoblast induction medium. This ensured that both the cytoplasm and nuclear localization could be easily visualised. Following fixation, cells were blocked and permeabilised (1 % FBS, 0.1 % Triton X-100 in PBS) before overnight incubation with ZNF25 antibody. Anti-rabbit alexa-fluor 488 (Invitrogen) was utilized as a secondary antibody and cells were counterstained with Phalloidin pre-conjugated with TRITC (5nM, Sigma) and Hoechst H33342 (0.1ug/ml, Sigma). Image acquisition was performed on a Perkin Elmer Operetta High Content Imaging System.

### Matrix mineralisation assay

Deposition of hydroxyapatite was measured using the OsteoImage™ Bone Mineralization Assay (Lonza) according to manufacturer’s instructions. Briefly, cells were plated in 96 well plates at 20,000/cm^2^ and induced in osteoblast induction medium for 15 days with media changed every third day. Following fixation (4 % formalin for 10 min at RT), wells were washed in Lonza wash buffer before staining with OsteoImage^TM^ staining reagent conjugated to 488 for 30 min at RT. Post-staining, wells were washed in wash buffer before being read on a FLUOstar Omega plate reader set at 488 nm emission wavelength.

### In vivo heterotopic bone formation

hMSC-TERT (0.5 × 10^6^) were suspended into single cells and combined with 40 mg hydroxy-apatite tricalcium phosphate as previously reported (HA/TCP, 0.5–1 mm granules, Biomatlante/Zimmer, Vigneux de Bretagne, France) [19–21]. Non-induced cells were incubated overnight in HA/TCP before implantation into the dorsolateral area of immune compromised mice (NOD.CB17-*Prkdc* ^*scid*^/J) for 8 weeks. After retrieval, implants were fixed overnight in 4 % formalin, washed in PBS before decalcification in formic acid for 3–5 days. Following embedding in wax, four serial sections were cut at three depths with 100um between each group and sections from each group were stained with haematoxylin and eosin, or human specific-vimentin antibody (AbCam).

### siRNA-based knock down experiments

LNA-modified Silencer® Select siRNAs targeting the desired genes (*ZNF608, ZBTB38* and *ZNF25*) and non-targeted negative controls 1 and 2 were purchased from Ambion (Invitrogen). Validation of siRNA data was done using a second Silencer® Select siRNA for *ZBTB38* and *ZNF608*, and one Mission siRNA (Sigma-Aldrich) for *ZNF25*. Reverse transfection of siRNA was performed using LipofectamineTM 2000 (Invitrogen) according to the manufacturer’s instructions. siRNA transfections were carried at as described in [3, 16].

### Affymetrix microarray gene expression analysis

hMSC-TERT cells were cultured and induced to differentiate into osteoblasts as described [20]. At 0, 3, 6, 9 and 12 days after induction, total RNA was extracted using TRIzol (Invitrogen) as previously reported [22]. Five hundred ng of total RNA from each sample were used for biotin-labeled cRNA production using a linear amplification kit (Ambion). First- and second-strand cDNA syntheses were performed from 8 μg total RNA using the SuperScript Choice System (Life-Technologies, Carlsbad, CA, USA) according to the manufacturer’s instructions. Subsequent hybridization and scanning of the Affymetrix arrays were performed as described previously [23]. The biotinylated targets were hybridized to HuGene 1.0ST v 1 Affymetrix oligonucleotide arrays. Expression measures were generated and normalized using the RMA procedure implemented in the Partek Genomics Suite version 6.12.0307. Values were then log2 transformed before further analysis. Affymetrix HuGene 2.0ST arrays were used for si*ZNF25* knock down and corresponding control samples. Partek Genomics Suite version 6.6 was used to analyse the resultant microarray data.

### Illumina bead chip microarray

hMSC-TERT cells were cultured and induced to differentiate into osteoblasts as described [20]. At days 0, 1, 7 and 13 after induction, total RNA was extracted from each of three independent cell cultures. At 90–100 % confluence, highly purified total cellular RNA was isolated using an RNeasy Kit (QIAGEN Nordic, West Sussex, UK) according to the manufacturer’s instructions. A total of 500 ng of total RNA from each sample was used for biotin-labeled cRNA production using a linear amplification kit (Ambion).

Hybridization, washing, Cy3-streptavidin staining and scanning were performed on the Illumina BeadStation 500 platform (Illumina) according to the manufacturer’s instruction. cRNA samples were hybridized onto Illumina HT12 V4 BeadChips. Analyses of gene expression data were carried out using the GenomeStudio software (v2011.1). Raw data were normalized using the quantile normalisation and then filtered for significant expression on the basis of negative control beads. A *p*-value of < 0.01 was used as a cut-off for detection of significance.

Differential gene expression analysis and significance testing was done using the ‘lumi’ BioConductor package [24]. After being checked for quality, data was transformed using the ‘variance stabilizing transform’. Data was quantile normalized and probes that passed the detection *p*-value threshold (*p* < 0.01), for at least one time point, were selected for further analysis. Differentially expressed genes were identified by a 2-way ANOVA, and Benjamini-Hochberg multiple testing correction.

### Extraction of curated transcription factors

Transcription factors were extracted from Illumina and Affymetrix datasets using the list of 1391 curated TFs detailed in [14]. This was done using Partek Genomics Suite (v 6.12.0307). Some TFs on the list were updated to their current Ensembl Identifiers. A small number of TFs were not present on the Affymetrix Gene ST array, and were thus excluded from the analysis.

### Self organising map-based cluster analysis

Self organising map (SOM) cluster analysis was performed using Partek Genomics Suite (v 6.12.0307). For the Illumina array dataset, the average gene expression of the three replicates at each time point was used as the gene expression measure. Gene expression data was shifted to a mean of zero before cluster generation to aid in the viewing of cluster profiles. The number of clusters in each map was varied between 25 and 81, in order to identify the optimum number of clusters for a particular dataset. The optimum number of clusters was achieved when each cluster displayed a single gene expression trend across the time course. For the Affymetrix array dataset, this was 64 clusters and for the Illumina array dataset it was 36.

### RNA isolation and real-time quantitative PCR

Total RNA was isolated using TRIzol (Invitrogen) as previously reported (15). For real-time quantitative PCR, data were normalised to the geometric mean of four reference genes (*β-Actin, B2M, HPRT, UBC1*) and analysed using a comparative Ct method. Primer sequences were designed using the Primer-BLAST tool (http://www.ncbi.nlm.nih.gov/tools/primer-blast/). Primer sequences are listed below in Table 1.

**Table 1:** Forward and reverse primer sequences for real-time quantitative PCR assays

Gene	Forward primer 5’-3’	Reverse primer 5’-3’
*ZNF25*	CCTGGGGCTGCCAGCTAAGGT	CAGGGAAGCCCCGATGTGGAA
*ZBTB38*	CACAGAAGCCCTCTAGCCAAG	AGCAGGAAAGCCCTCCTAGA
*ZNF608*	GTGGTCAATGTCACGTGGAG	AGCCCTCTGGACTCTGTGAA
*RUNX2*	TGGTTACTGTCATGGCGGGTA	TCTCAGATCGTTGAACCTTGCTA
*COL1A1*	AGGGCTCCAACGAGATCGAGATCCG	TACAGGAAGCAGACAGGGCCAACGTCG
*ALPL*	ACGTGGCTAAGAATGTCATC	CTGGTAGGCGATGTCCTTA
*BGLAP*	CATGAGAGCCCTCACA	AGAGCGACACCCTAGAC

### Western blot analysis

Western blot analyses were carried out on control and differentiated OB cells as previously reported [20]. The antibodies used were ZNF25 (Novus Biologics antibody H00219749-B01) and alpha-tubulin (Sigma-Aldrich) whereas goat anti Rabbit IgG-HRP (SantaCruz Biotechnology, Inc, Heidelberg, Germany) was used as the secondary antibody.

### BLAST analysis and domain alignment

Evolutionary analysis of ZNF25 protein sequence was performed using the blastp program available at http://blast.ncbi.nlm.nih.gov/Blast.cgi. Domain alignments of ZNF25 orthologs were generated using the ‘Illustrator for Biological Sequences’ tool (version 1) [25].

## Results

### hMSC-TERT differentiate into osteoblastic cells in vitro and form heterotopic bone in vivo

**Fig. 1:**
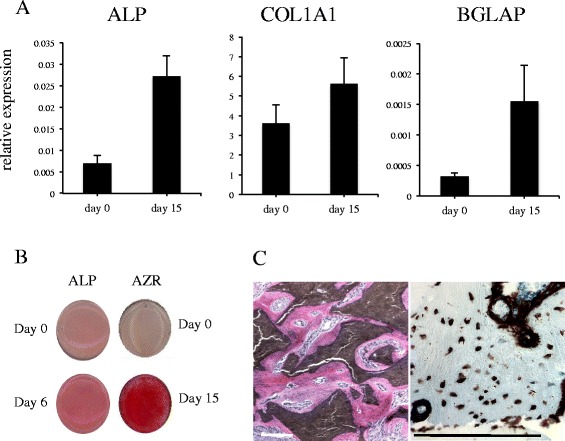
hMSC-TERT differentiate into osteoblastic cells in vitro and form heterotopic bone in vivo. **a** RT-PCR experiments show gene expression of canonical osteoblastic markers ALP, COL1A1 and BGLAP in hMSC-TERT cells at day 0 and day 15 post-induction of OB differentiation. The error bars (S.E.M.) represent 4 independent biological replicates. The y axis represents expression relative to a set of housekeeping genes. **b** Alkaline phosphatase (ALP) activity in hMSC-TERT cells at days 0 and 6 post-OB induction. Alizarin *red* (AZR) staining for matrix mineralisation at days 0 and 15 post-OB induction. **c** In vivo bone-formation assay was performed by implanting hMSC-TERT, mixed with hydroxyapatite–tricalcium phosphate (HA/TCP), subcutaneously into immune-deficient mice for 8 weeks. Sections were stained with hematoxylin (*pink* osteoid) and eosin (*blue* nuclei) (*left hand image*) or human- specific vimentin demonstrating human origin of osteoid and osteocytes (*right hand image*). The scale bar =100 μm

hMSC-TERT differentiate readily into osteoblastic cells, evidenced by enhanced expression of osteoblast marker genes (*ALPL, BGLAP, COL1A1*) (Fig. 1a), increased alkaline phosphatase activity and formation of in vitro mineralized matrix stained positive for Alizarin red during differentiation (Fig. 1b). hMSC-TERT cells are also able to form heterotopic bone when implanted subcutaneously, with hydroxyapatite tricalcium phosphate (HA/TCP), in immune deficient mice (Fig. 1c).

### Transcription factors associated with in vitro osteoblast differentiation

Two microarray datasets were generated during in vitro osteoblast differentiation of hMSC-TERT: Illumina HT12-v4 arrays at four time points (days 0, 1, 7, 13) and Affymetrix HuGene 1.0 STv1 arrays at six time points (days 0, 3, 6, 9, 12). These datasets will be referred to as the ‘Illumina’ and ‘Affymetrix’ datasets. The Illumina time course dataset was filtered to extract expression patterns for 1391 Human TFs, as curated by Vaquerizas et al. [14]. This produced a list of 1141 probes, mapping to 738 unique TFs. One hundred and forty nine TFs showed expression changes of 0.71 > FC > 1.40 between day 0 and day 13 post OB induction, with an adjusted *p* value of 0.20. This threshold was used because *RUNX2* and *ATF4*, the major regulators of osteoblastogenesis, displayed a fold change of 1.48 (p adj = 0.141, p unadj = 0.048) and 1.48 (p adj = 0.18, p unadj = 0.065) in the Illumina dataset, respectively. For the Affymetrix dataset, 1380 TFs were extracted, using the same curated set from Vaquerizas et al. [14].

### Transcription factors cluster according to temporal expression during in vitro osteoblast differentiation

**Fig. 2:**
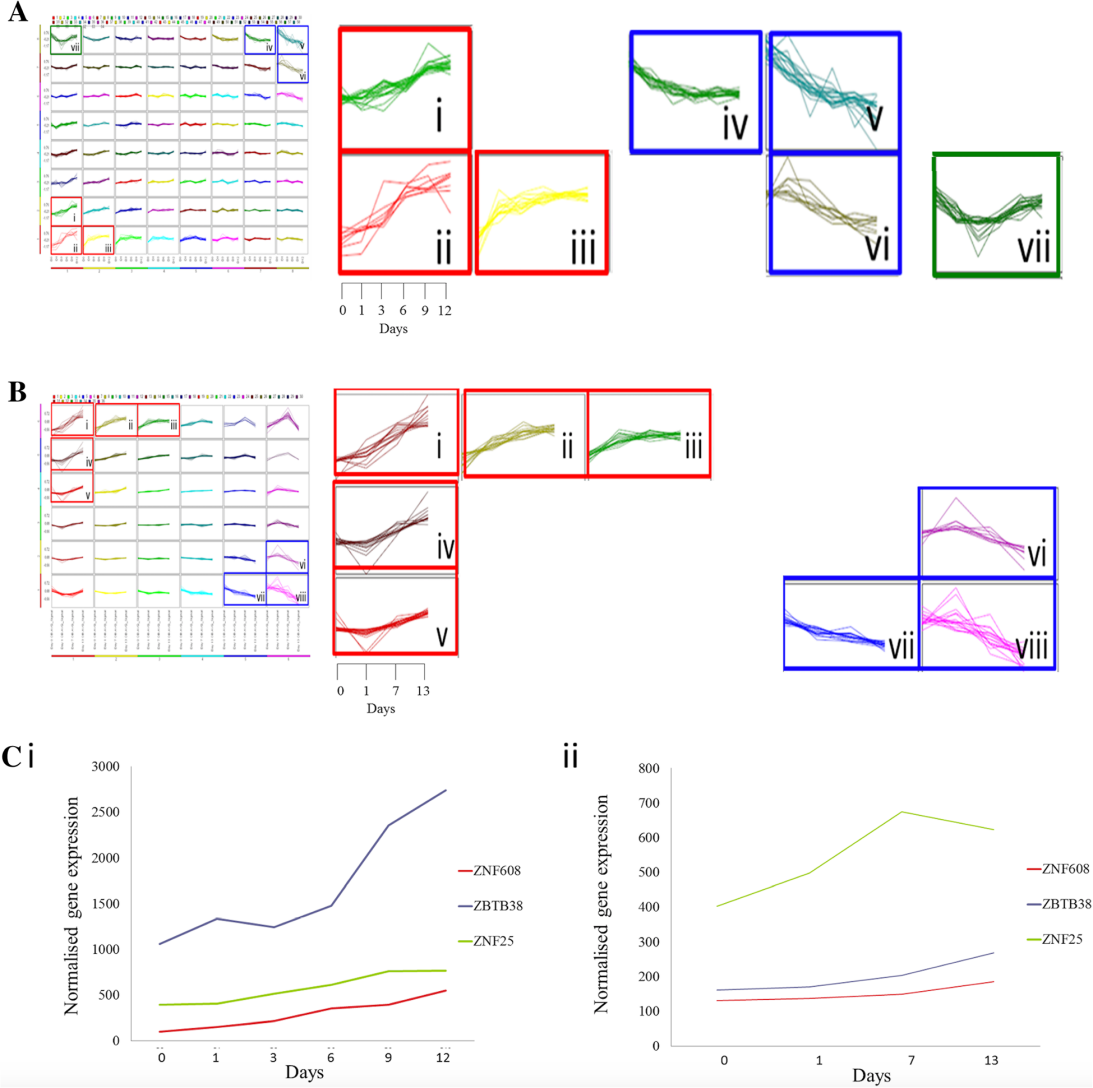
Transcription factor expression profiles across an osteoblast differentiation time course. **a** Transcription factor expression levels were extracted from Affymetrix microarrays and clustered with self organizing maps. The Y axis of each cluster cell is normalised, log2 gene expression, while the X axis displays the six time points in days (day 0,1,3,6,9,12). Clusters displaying a distinct change in expression profile across time are outlined in the map and shown to the right. There are three upregulated clusters, outlined in *red*: i (17 genes), ii (8 genes) and iii (13 genes). There are three downregulated clusters, outlined in *blue*: iv (16 genes), v (17 genes) and vi (9 genes). The cluster (vii), outlined in *green*, contains 18 genes and showed a decrease in expression during early differentiation, followed by an upregulation in expression from day 3 to day 12. **b** Transcription factor expression levels were extracted from Illumina microarrays and clustered with self organizing maps. The Y axis of each cluster cell is the normalised, log2 gene expression, while the X axis displays the four time points in days (day 0,1,7,13). Clusters displaying a distinct change in expression profile across time are outlined. These clusters are expanded in the right hand side of the image. There are five upregulated clusters, labelled i (14 genes), ii (15 genes), iii (17 genes), iv (13 genes) and v (18 genes), outlined in *red*. There are three downregulated clusters labelled vi (9 genes), vii (15 genes) and viii (16 genes) and these are outlined in *blue*. **c** Gene expression profiles across the OB differentiation time-course for transcription factors *ZNF25, ZBTB38 and ZNF608*. These are extracted from the (i) Affymetrix array data and (ii) Illumina array data. The time points in days post-OB induction are labeled on the X axis, while the Y axis displays normalized gene expression values. For the Affymetrix array plot (i), the fold change values between day 12 and day 0 are: *ZNF25* (1.9 FC), *ZBTB38* (2.6 FC) and *ZNF608* (5.3 FC). For the Illumina array plot (ii), the fold change and *p* values are for day 13 versus day 0: *ZNF25* (1.55 FC, *p* = 0.067), *ZBTB38* (1.66 FC, *p* = 0.025) and *ZNF608* (1.41 FC, *p* = 0.0013). The Illumina array TF plot (ii) displays an average of the 3 biological replicates at each time point

To identify groups of genes that showed similar temporal expression patterns during in vitro osteoblastic differentiation, self-organizing maps (SOMs) were used (Fig. 2). For the Affymetrix TF dataset, six clusters showed distinct up or down regulation across the time-course (Fig. 2a). The up-regulated clusters i, ii and iii contained 17, 8 and 13 genes respectively whereas the down-regulated clusters iv, v and vi contained 16, 17 and 9 genes, respectively. Interestingly, another cluster showed a decrease in gene expression from day 0 to day 3, followed by an increase in gene expression to day 12. The TFs contained within all clusters are listed in Table 2.

**Table 2:** Members of transcription factor clusters identified during osteoblast differentiation, derived from the Affymetrix dataset

Upregulated clusters			Downregulated clusters			V-shaped cluster
Cluster i	Cluster ii	Cluster iii	Cluster iv	Cluster v	Cluster vi	Cluster vii
*AHR*	*EPAS1*	*BCL6*	*AFF3*	*DLX1*	*OSR1*	*BACH1*
*DDIT3*	*HES1*	*EBF1*	*ARNTL2*	*DLX2*	*RARB*	*BHLHE40*
*EGR2*	*NR4A3*	*FOXO1*	*CREB3L2*	*E2F1*	*SMAD9*	*CEBPG*
*RORA*	*PRDM1*	*KLF15*	*CTCFL*	*EGR1*	*TSHZ1*	*CREB5*
*SALL4*	*RUNX2*	*KLF5*	*DRAP1*	*EZH2*	*DPF1*	*ETV1*
*ZBTB1*	*STAT4*	*MKX*	*FOSL1*	*FOXM1*	*TFAP2A*	*ETV4*
*ZBTB38*	*ZBTB16*	*NFIA*	*LHX9*	*GATA2*	*RARG*	*ETV5*
*ZEB2*	*ZNF608*	*NPAS2*	*MXD3*	*ID1*	*ZNF93*	*FOXQ1*
*ZNF117*		*OSR2*	*NFATC4*	*KLF4*	*ZNF519*	*HIVEP2*
*ZNF235*		*SIX1*	*PAX6*	*MEF2C*		*JUN*
*ZNF25*		*STAT2*	*PRDM16*	*MYBL1*		*NR1D1*
*ZNF345*		*THRB*	*RFX2*	*MYBL2*		*NR1D2*
*ZNF354A*		*TSC22D3*	*TP63*	*SNAI1*		*TBX3*
*ZNF385A*			*WHSC1*	*SOX9*		*TFAM*
*ZNF449*			*ZNF90*	*TCF19*		*ZNF295*
*ZNF493*				*TCF7*		*ZNF326*
*ZNF566*				*ZNF695*		*ZNF643*
						*ZNF678*

We examined all the clusters containing upregulated genes, for the presence of TFs that have known roles in osteoblast differentiation. Cluster *i* contained 17 TFs, of which seven have been previously documented to be involved in osteoblastogenesis in mouse and rat models. These include *AHR, DDIT3, EGR2, RORA, SALL4, ZEB2 and ZNF385A* [26] [27–30]. Cluster *ii* contained *RUNX2*, along with TFs *EPAS1, HES1,NR4A3, PRDM1, STAT4* and *ZBTB16*; these are known to either be involved in osteoblastic differentiation or bone homeostasis, as direct regulators or via an interaction with a regulator [31–36]. Cluster *iii* contained 13 TFs, all of which have previously been documented to be involved in hMSC differentiation into either osteoblasts, adipocytes or chondrocytes (e.g. *BCL6, EBF1, FOXO1, KLF15, KLF5, MKX, NFIA, NPAS2, OSR2, SIX1, STAT2, THRB* and *TSC22D3)* [13] [37–44].

We further examined the upregulated clusters for TFs that are currently not associated with differentiation and development. This revealed a total of 11 TFs of interest; ten of which were in cluster *i*. The majority of these are zinc finger proteins (*ZNF25, ZNF117, ZNF235, ZNF345, ZNF354A, ZNF 449, ZNF493, ZNF566*) and two are zinc finger and BTB domain-containing proteins, *ZBTB1* and *ZBTB38.* The final TF was in cluster *ii*, which was zinc finger protein *ZNF608*.

The Illumina TF dataset was also subjected to the same process of SOM clustering as described for the Affymetrix dataset. In this case, this produced 36 clusters of TF expression profiles. Each cluster contained between 3 and 44 genes. Five clusters showed up-regulation of TFs across the time-course (labeled *i-v* in Fig. 2b), while three clusters showed a distinct down-regulation (labeled *vi, vii* and *viii* in Fig. 2b). The TF content and gene expression information of each labeled cluster is detailed in Table 3. The total of 77 TFs in clusters *i* to *v* were examined to identify which TFs have previously been implicated in MSC differentiation, bone formation or related mesodermal differentiation processes such as adipogenesis. Fifty nine of the TFs were found to be differentiation-associated, whereas 16 TFs were identified as potentially novel osteoblast-differentiation associated TF candidates. These included *ZNF181, ZNF697, ZNF295, ZNF22, ZNF532, ZNF302, ZNF217, ZNF721, ZNF25, ZNF608, ZNF419, ZNF558 and ZNF627,* along with two zinc finger- and BTB-domain containing proteins *ZBTB38* and *ZBTB40* and zz-type zinc finger-containing protein *ZZZ3.*

**Table 3:** Members of transcription factor clusters identified during osteoblast differentiation, derived from the Illumina dataset

Upregulated clusters					Downregulated clusters		
Cluster i	Cluster ii	Cluster iii	Cluster iv	Cluster v	Cluster vi	Cluster vii	Cluster viii
*ZBTB16*	*AHR*	*CDC5L*	*ZBTB38*	*ZNF181*	*E2F4*	*SOX9*	*TFAP2A*
*KLF9*	*FOXN2*	*SNAI2*	*BMP2*	*ZNF697*	*ID3*	*DLX1*	*MYBL1*
*EPAS1*	*TSC22D3*	*TGIF1*	*FOXC1*	*ATF4*	*ZNF207*	*HOXB2*	*ELF4*
*FOXO1*	*ZFP36L1*	*FOS*	*CEBPG*	*JUN*	*NFIC*	*NFIX*	*ID2*
*MYC*	*NFIA*	*STAT2*	*E2F5*	*MXD1*	*TSHZ1*	*PITX1*	*E2F2*
*SOX4*	*NFE2L2*	*NFIB*	*EGR1*	*NR1D2*	*MXD4*	*SRF*	*DRAP1*
*STAT4*	*FOSB*	*NR2F1*	*DDIT3*	*FOXJ3*	*PRRX2*	*TEAD4*	*TFDP1*
*CEBPB*	*ZNF22*	*CEBPZ*	*MAFB*	*SATB2*	*ZNF503*	*NR2F6*	*ATF5*
*NFIL3*	*HBP1*	*SMAD5*	*ZEB2*	*TBX3*	*EZH2*	*NFE2*	*KLF2*
*IFI16*	*SIX4*	*ZNF217*	*ZBTB40*	*MYNN*		*TCF25*	*SOX18*
*NR4A2*	*ZNF532*	*RERE*	*ZZZ3*	*ZNF608*		*ZNF668*	*ZNF395*
*BATF*	*EBF1*	*ZBTB20*	*ZNF295*	*ZNF419*		*ZNF408*	*MXD3*
*ZBTB33*	*ELF1*	*BBX*	*MKX*	*ZNF622*		*ZNF672*	*OSR1*
*FOXQ1*	*HIF1A*	*ZNF462*		*ZNF558*		*ZNF511*	*ID1*
	*ZNF302*	*ZNF721*		*ZNF627*		*CREB3L2*	*THRA*
		*ZNF25*		*TBX15*			*FOXM1*
		*PRDM1*		*TCF12*			
				*KLF11*			

### Identification of three novel TFs associated with in vitro osteoblast differentiation

**Fig. 3:**
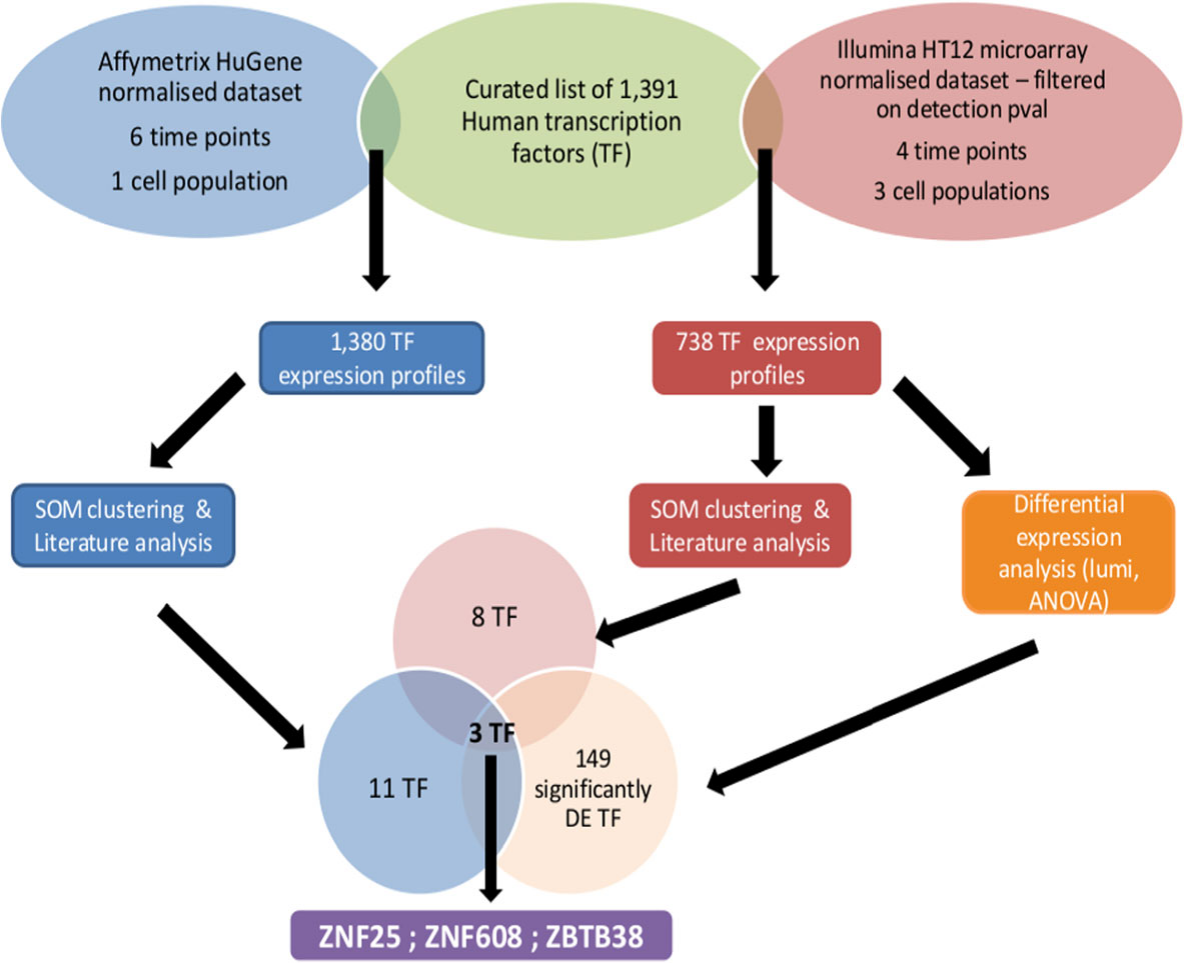
The method used to identify ZNF25, ZNF608 and ZBTB as candidates of interest in osteogenesis. The gene expression profiles for a number of transcription factors were extracted from two microarray datasets (Illumina and Affymetrix), using a curated set of human transcription factors (TFs). Self Organising Maps (SOMs) were then used to cluster the subsets of TFs according to temporal expression pattern. TFs that were upregulated in SOM clusters were then further screened based on literature analysis. Upregulated TFs that were previously unreported to be associated with hMSC differentiation, bone formation or related processes were selected for further analysis. In addition, the TF expression profiles extracted from the Illumina array dataset were analysed for differential expression. TFs that were significantly differentially expressed between undifferentiated hMSC and differentiated OB were selected for further analysis. Three TFs satisfied all of these selection criteria, namely *ZNF25*, *ZBTB38* and *ZNF608*

**Fig. 4:**
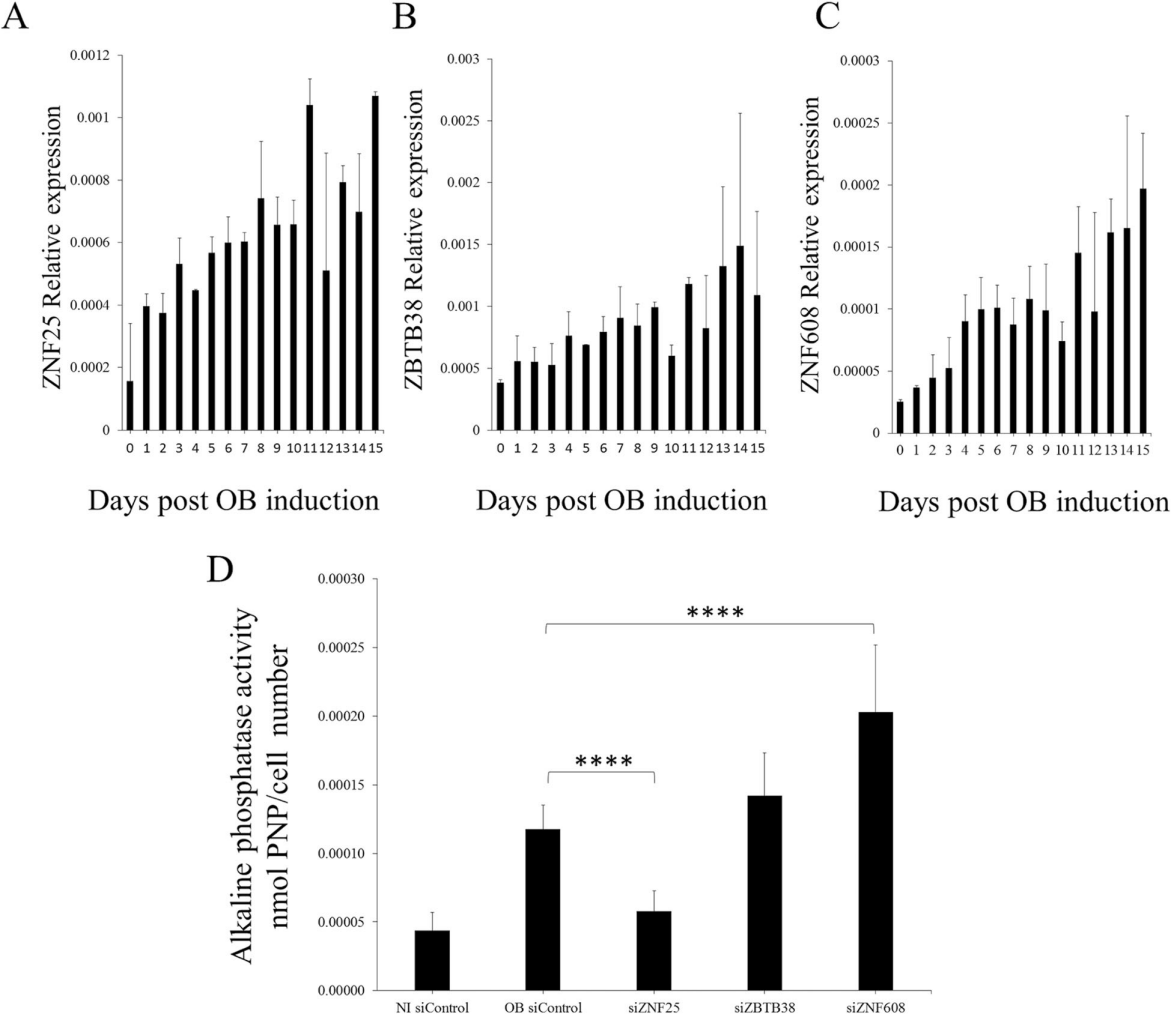
Validation of expression increases for *ZNF25, ZBTB38* and *ZNF608* during OB differentiation in hMSC-TERT cells. **a**-**c** Quantitative RT-PCR showing expression of *ZNF25, ZBTB38* and *ZNF608* in hMSC-TERT cells during OB differentiation*.* The Y axis represents mean expression relative to a set of housekeeping genes (refer to Methods). The X axis represents days post-induction of OB differentiation. Error bars reflect SEM from 4 biological replicates. **d** Alkaline phosphatase activity in hMSC-TERT cells after siRNA knockdown of TFs *ZNF25, ZBTB38* and *ZNF608*. The alkaline phosphatase activity was measured at day 6 post-OB induction for all samples, where the y axis reflects units of P-nitrophenylphosphate normalised to cell number. The NI siControl refers to hMSC-TERT cells not induced for OB differentiation and transfected with a scrambled control siRNA. The OB siControl refers to hMSC-TERT cells induced for OB differentiation and transfected with a scrambled control siRNA. A highly significant reduction in ALP activity was observed between si*ZNF25* and OB siControl (*p* = 7.89 × 10^−7^) and a highly significant increase in ALP activity was observed between si*ZNF608* and the OB siControl (*p* = 1.68 × 10^−6^); these are marked by four asterisks. There was no significant change in alkaline phosphatase activity between si*ZBTB38* and OB siControl

We employed a number of criteria to select TFs for further analysis. As shown in Fig. 3, the TFs had to be present in up-regulated SOM clusters in both Affymetrix and Illumina analyses, the TFs had to be previously unreported in association with hMSC differentiation, bone formation or related processes and, finally, the TFs had to exhibit a statistically significant increase in expression during in vitro OB differentiation. Three TFs satisfied these criteria, namely zinc finger protein 25 (*ZNF25*), zinc finger- and BTB-domain containing protein 38 (*ZBTB38*) and zinc finger protein 608 (*ZNF608*). The fold change increase in gene expression and *p*-values showed by these during differentiation for day 13 versus day 0 were *ZNF25* (1.55 FC, *p* = 0.067), *ZBTB38* (1.66 FC, *p* = 0.025) and *ZNF608* (1.41 FC, *p* = 0.0013) in the Illumina dataset (Fig. 2c). We further validated gene expression profiles of *ZNF25, ZNF608 and ZBTB38*, employing quantitative RT-PCR in independent biological experiments (Fig. 4a–c). Similar to the microarray data, all three TFs displayed a temporal increase in gene expression with maximal expression during the late phase (days 10–15) of in vitro osteoblast differentiation (Fig. 4a–c). There was also a statistically significant difference in qPCR expression measurements between day 0 and day 15 for ZNF25 and ZNF608. However, ZBTB38 did not display a significant difference. (Student’s *t*-test, *ZNF25 p* = 0.0027, *ZNF608 p* = 0.0089 and *ZBTB38 p* = 0.3353).

### siRNA knock-down of ZNF25 affects osteoblast differentiation

The temporal gene expression pattern of the candidate TFs suggested a role in osteoblast differentiation of hMSC. To test this hypothesis, we examined the effects of siRNA-mediated knockdown of each of the TFs on ALP activity, as a marker of the osteoblastic phenotype. A statistically significant decrease in ALP activity was seen on knockdown of *ZNF25* (*p* < 0.005, Fig. 4d), relative to that of the scrambled siRNA control. By contrast, siRNA-mediated knockdown of *ZNF608* and *ZBTB38* did not show a reduction on ALP activity. On the other hand, knockdown of *ZNF608* actually showed a statistically significant increase (*p* = 0.00002) in ALP activity. For the purposes of this study we further focused our analyses only on *ZNF25*.

### ZNF25 protein expression increases during osteoblast differentiation

**Fig. 5:**
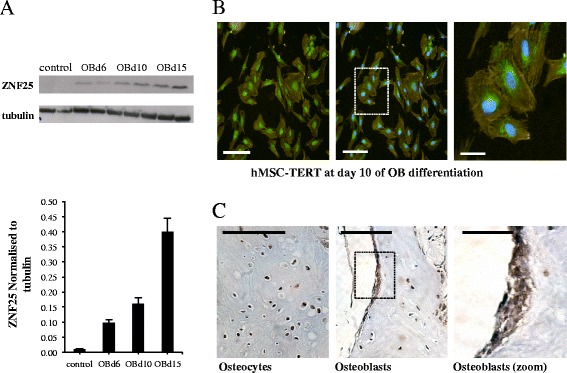
Expression and localisation of the ZNF25 protein. **a** Protein levels of ZNF25 increase during OB differentiation of hMSCs, as revealed by western blotting and quantitation of the same blot. **b** ZNF25 is localised to the nucleus and perinuclear area after immunocyto-chemical staining during OB differentiation. *Left*, middle and right images show hMSC-TERT cells at day 10 of OB differentiation; ZNF25 was detected by immunostaining (*green*) (*left image*), cells were counterstained with Phalloidin pre-conjugated with TRITC (*yellow/orange*, cytoplasmic actin) and Hoechst H33342 (*blue*, nucleus) (*middle and right image*). This confirms the localisation of ZNF25 to the nucleus and perinuclear region as highlighted in the right image. The *dotted rectangle* in the middle image indicates area that is magnified to form the right image. The *white scale bars* in the bottom left of the images indicate a distance of 100 μm in left and middle image, and 30 μm in the right image. **c** Antibody-based localisation of ZNF25 in the human femoral neck bone demonstrates highly positive *brown* staining in the osteocytes (*left*) and in osteoblast surfaces (middle and in higher magnification on right). The *dotted rectangle* in the middle image indicates the area that is magnified to form the right image. There is a small amount of non-specific staining in other cell types in this image. The *black scale bars* in the top left of the images indicate a distance of 50 μm for the left and middle images, and 16 μm for the right image

We investigated the temporal expression of the ZNF25 protein and its localization in cells and in human bone tissue biopsies. Similar to *ZNF25* gene expression, the production of the ZNF25 protein was found to increase during in vitro osteoblast differentiation, at days 6, 10 and 15 (Fig. 5a). In keeping with its role as a transcription factor, in vitro staining of hMSC-TERT revealed it to have nuclear and perinuclear localization (Fig. 5b). In human femoral neck bone biopsies, immunostaining localized the ZNF25 protein to active osteoblastic surfaces as well as osteocytes (Fig. 5c).

### ZNF25 siRNA knockdown causes increase in expression of genes relevant to osteoblast differentiation

**Fig. 6:**
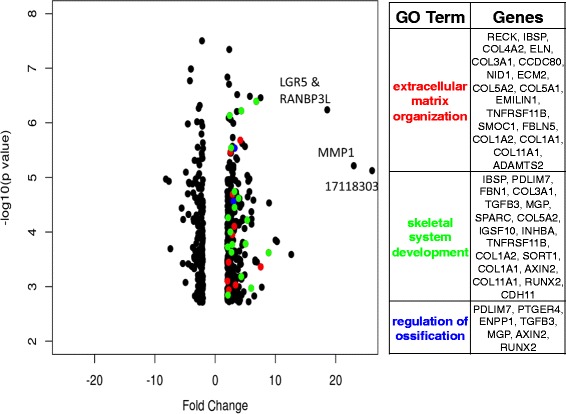
ZNF25 siRNA knockdown increases the expression of MMP1, LGR5, RANBP3L and other osteoblastic differentiation genes. Volcano plot representing the 512 genes most differentially expressed between siZNF25 knockdown and control hMSCs at day 14 post-OB induction. The siZNF25/control fold change for each gene is represented on the x axis, with the corresponding p value represented on the y axis (expressed as –log(*p* value)). Genes which have the Gene Ontology (GO) annotation ‘extracellular matrix organisation’ are coloured in *red*. Genes with GO annotation ‘skeletal system development’ are coloured in *green* and those with GO annotation ‘regulation of ossification’ are coloured in *blue*. Genes in these three categories are listed on the right. This figure represents ‘extracellular matrix organisation’, ‘skeletal system development’ and ‘regulation of ossification’ annotation categories only, as genes with the annotations ‘ossification’ and ‘bone development’ are all contained within the other 3 categories. There were four very highly upregulated genes (labelled on the plot). These are matrix metallopeptidase 1 (MMP1, FC = 23.00, pval = 6.09 × 10^–6^), leucine-rich repeat containing G protein-coupled receptor 5 (LGR5, FC = 18.59, pval = 5.77 × 10^–7^), RAN binding protein 3-like (RANBP3L, FC = 18.08, pval = 1.81 × 10^–9^) and unannotated transcript cluster, 17118303 (FC = 26.05, pval = 7.49 × 10^–6^). On further investigation it was found that this latter transcript cluster maps to chromosomal location: chr7:94058513–94060553, which corresponds to exon 52 of the COL1A2 gene. LGR5 and RANBP3L have very similar expression values and so the dots on the volcano plot for each gene are overlapping

ZNF25 contains a KRAB domain, and is therefore likely to act as a transcriptional repressor [45]. To gain insight into the possible targets of *ZNF25* and confirm its repressor activity, we used microarrays to analyse the gene expression of control and *ZNF25* knockdown cells (si*ZNF25*) at days 0 and day 14, post-osteoblast differentiation (Fig. 6). At day 0, 50 genes were identified as differentially expressed between si*ZNF25* and control samples (3 upregulated and 47 downregulated using two - fold change and FDR <0.05 as threshold). At day 14, by contrast, 520 genes were identified as differentially expressed between si*ZNF25* and control samples (347 upregulated, 173 downregulated and using two - fold change and FDR < 0.05 as threshold). Since *ZNF25* contains a KRAB domain and is likely to be a transcriptional repressor, any genes that show marked upregulation could be of functional significance. At day 0, there were no genes that showed dramatic upregulation in si*ZNF25* cells. However at day 14 there were four genes that showed 18-fold to 26-fold upregulation. The most significantly upregulated gene in differentiated si*ZNF25* cells relative to control was matrix metallopeptidase 1 (*MMP1*, FC = 23.00, pval = 6.09 × 10^−6^). The next most upregulated genes were leucine-rich repeat containing G protein-coupled receptor 5 (*LGR5*, FC = 18.59, pval = 5.77 × 10^−7^) and RAN binding protein 3-like (*RANBP3L*, FC = 18.08, pval = 1.81 × 10^−9^). We also observed a very high upregulation in unannotated transcript cluster, 17118303 (FC = 26.05, pval = 7.49 × 10^−6^). On further investigation it was found that this transcript cluster maps to chromosomal location: chr7:94058513–94060553. This chromosomal location corresponds to exon 52 of the *COL1A2* gene, however no other probes for this gene were upregulated to this level.

DAVID functional enrichment analysis [46, 47] of the 520 genes differentially expressed at day 14 post-OB induction showed enrichment for: ‘extracellular matrix organisation’ (*p* value = 2.9 × 10^−11^, FDR = 4.6 × 10^–8^), ‘ossification’ (*p* value = 2.08 × 10^−5^, FDR = 0.035), ‘bone development’ (*p* value = 3.9 × 10^−5^, FDR = 0.06), ‘skeletal system development’ (*p* value = 2.69 × 10^−4^, FDR = 0.459) and ‘regulation of ossification’ (*p* value = 0.0049, but with a higher FDR of 8.08). The genes involved in these enrichment categories are all upregulated (Fig. 6). Analyses of the same gene sets by ReviGO [48] and GOrilla [49] yielded functionally similar results (data not shown). Background used for all enrichment analyses was the Affymetrix Human Gene 2.0 ST array gene set.

### ZNF25 is conserved but found only in tetrapod vertebrates

**Fig. 7:**
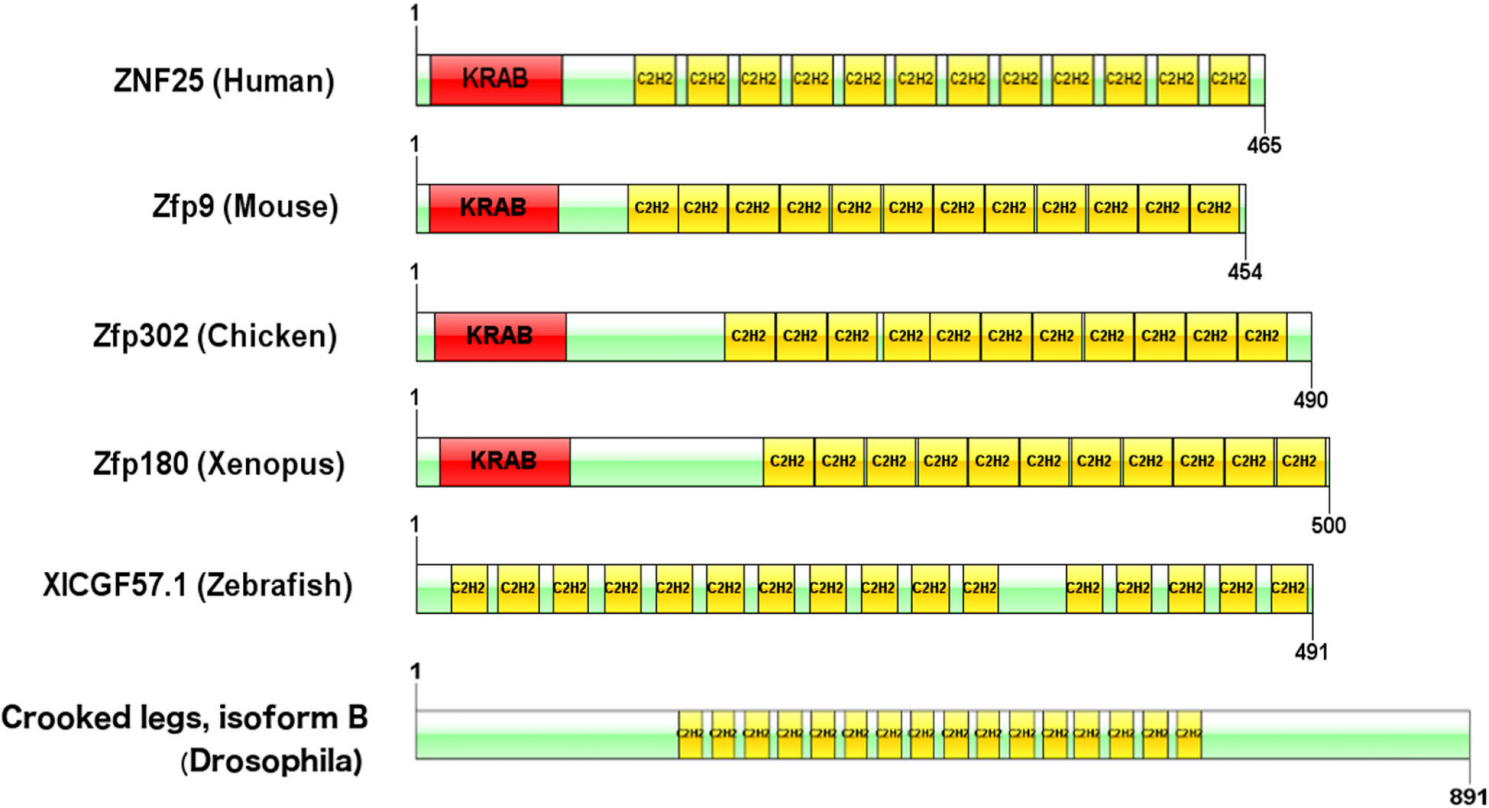
Domain alignment between ZNF25 and its homologs in mouse, chicken and xenopus. These are drawn approximately to scale. There were no direct orthologs discovered in Zebrafish or in Drosophila. However the closest matching proteins in these species, that contain multiple C2H2 domains, are shown. The KRAB domain is coloured in *red* and C2H2 domains are coloured in *yellow*. The length of each protein is denoted by numbers at the start and end the protein. The Drosophila crooked legs protein is larger and is not drawn to the same scale

The evolutionary conservation of *ZNF25* was then investigated. ZNF25 was highly conserved amongst mammals, with the top 250 top-ranked proteins from BLAST analysis being from mammalian species. The mouse protein ZFP9, for example, showed 79 % sequence identity with an E value = 0.0 and appeared as a true ortholog of ZNF25 via a reciprocal BLAST with all human proteins. Use of the GABLAM tool [50] also identified ZNF25 mammalian homologs in seven species. These observations are consistent with results in the NCBI homologene database, that details ZNF25 orthologs for mammalian species, including *Pan troglodytes, Macaca mulatta, Canis lupus, Bos taurus, Mus musculus and Rattus norvegicus*. For other species, we observed full length matches to the chicken protein ZFP302 (44 % sequence identity, E value = 8 × 10^-126)^ and *Xenopus tropicalus* zinc finger protein 180 (44 % sequence identity, E value = 7 × 10^−137^). However these are unlikely to be true orthologs as they did not identify ZNF25 via reciprocal BLAST. Whilst a single KRAB domain was present in each protein, different numbers of C2H2 domains were present, with 12 in human but 11 in chicken and Xenopus. In zebrafish, there was partial homology to the ZNF25 C2H2 domain found in gastrula zinc finger protein XICGF57.1-like (49 % sequence identity, E value = 6 × 10^−126^). In the fruit fly, there was also partial homology to the ZNF25 C2H2 domain in the crooked legs protein, isoform B (44 % sequence identity, E value = 1 × 10^−86^). The KRAB domain was not present in these two proteins. These results indicate that the KRAB domain of ZNF25 protein is conserved in tetrapod vertebrates. Figure 7 shows a domain alignment of ZNF25 and homologs identified by the BLAST analysis.

## Discussion

This study has shown that a novel transcription factor, *ZNF25*, has a role in the differentiation of hMSCs to osteoblastic cells. We observed that expression of the transcription factor *ZNF25* increased steadily during OB differentiation of hMSCs, and its deficiency significantly decreased alkaline phosphatase levels in differentiating osteoblastic cells. The ZNF25 protein is present in the nuclei of mature osteoblastic cells and osteocytes in human bone. These results suggest that *ZNF25* plays a role in in osteoblast differentiation of hMSCs.

*ZNF25* belongs to the Krueppel C2H2-type zinc-finger protein family and contains 12 C2H2-type zinc fingers and one KRAB domain [30]. The Krueppel-associated box (KRAB) is a domain of around 75 amino acids that is found in the N-terminal part of about one third of eukaryotic Krueppel-type C2H2 zinc finger proteins (KRAB-ZFPs). These KRAB-ZFPs make up the largest family of zinc finger transcription factors in mammals and are only found in tetrapod vertebrates [51]. The KRAB-ZFP family has expanded greatly to include hundreds of members in mammals [52]. The KRAB domain acts as a transcriptional repressor by binding to corepressor proteins, whereas the C2H2 zinc-finger domains bind DNA. The function of proteins in the KRAB family include repression of RNA polymerase II and III promoters and binding and splicing of RNA [51].

KRAB-zinc finger proteins are known to play important roles during cell differentiation and development. Individual members of one subfamily of KRAB zinc finger genes (*ZNF91*) are restricted to specific hematopoietic cell lineages and may play a role in lineage commitment, possibly silencing transcription from nonlineage-expressed genes [53]. One of the other KRAB zinc finger proteins is involved in osteoblast differentiation. *AJ18* (*ZFP354C*) was identified by Jhoen et al. as a repressor of osteoblast differentiation in rat embryonic tibia and calvariae [54]. Overexpression of *AJ18* suppressed *RUNX2* activity and repressed the markers of osteoblast differentiation *ALP* and*, BGLAP*. Our BLAST analysis confirmed the KRAB domain is only found in tetrapod vertebrates. Previous studies have reported that KRAB-ZFPs have expanded to a large degree in mammals [52, 55].

The temporal expression profile of *ZNF25* clustered with profiles of other transcription factors known to be involved in osteogenesis. These transcription factors include *SMAD5, FOS,* and *SNAI2. SMAD5* functions synergistically with *SMAD1* and *RUNX2* to induce osteoblast-specific gene expression in C2C12 cells [56]. FOS proteins heterodimerize with JUN proteins to form the AP1 transcription factor complex, which is an important regulator of bone formation [57]*. SNAI2* binds to *RUNX2* promoters in vivo and acts as a positive transcriptional regulator in human osteoblasts [58]. Whilst the profile of *ZNF25* expression does not allow us to understand its exact timing in any transcription factor cascade, its co-expression with the transcription factors, above, emphasizes its association with ostoegenesis.

To discover putative genes that are targets of *ZNF25*, and thus suggest possible molecular mechanisms by which *ZNF25* affects osteoblast differentiation, we identified the genes that are differentially regulated following siRNA-mediated knock down. Given that *ZNF25* has a KRAB domain, and is likely to be a transcriptional repressor, we anticipated the upregulation of a number of osteogenic genes. *MMP1, RANBP3L* and *LGR5* showed striking upregulation and are functionally related to osteoblast differentiation.

MMP1 was initially described as a collagenase to degrade fibrillary collagen (type I, II and III) [59] and other extracellular matrix proteins: fibronectin, aggrecan, laminin, perlecan, and vitronectin [59]. MMP1 has also non-extracellular matrix substrates (pro-TNFa, IGF, SDF1a and MCP 1–4). Degradation of these substrates, such as COL1A1, by increased MMP1 may lead to impaired extracelluar matrix levels and/or organization. Since ECM abundance, structure and/or content may be important triggers for mineralization, this may explain the apparent impairment of in vitro formation of mineralized matrix. *RANBP3L* has recently [60] been reported to be a nuclear export factor for Smad1/5/8, which are effectors of canonical BMP signaling. Canonical BMP signalling is tightly regulated through reversible phosphorylation and nucleocytoplasmic shuttling of Smad1/5 and 8. Interestingly, Chen et al. [60] showed that overexpression of *RANBP3L* blocks BMP-induced osteogenesis of mouse BM-MSCs, while depletion of *RANBP3L* expression enhances BMP-dependent MSC differentiation activity and transcriptional responses. Further to this, the overexpression of *RANBP3L* in BM-MSCs was shown to result in reduced ALP activity and alizarin red staining, a phenotype which is consistent to what we observed on knockdown of *ZNF25* and its resulting increase in RANBP3L expression. However, Chen et al. [60] reported that *RANBP3L* overexpression compromised the BMP-induced expression of the preosteoblast marker *RUNX2*, whereas we did not detect any changes in expression of *RUNX2*. Thus it is likely that *RANBP3L* acts earlier in the differentiation process than *ZNF25. LGR5* is an orphan G-protein coupled receptor and is a direct target of canonical Wnt signaling. *LGR5* potentiates the canonical Wnt signaling pathway by binding to R-spondins [61]. *LGR5* also a mouse marker of stem cells in small intestine and colon [62]. Its relationship to osteoblast differentiation is currently unknown.

## Conclusions

In this study we have shown that the uncharacterized transcription factor, *ZNF25*, has a role in the differentiation of hMSCs to osteoblasts. ZNF25 appears to act as a transcriptional repressor via a KRAB domain and we identified three potential targets of ZNF25, matrix metallopeptidase 1, leucine-rich repeat containing G protein-coupled receptor 5 and RAN-binding protein 3-like. Future studies will determine the role of this transcription factor in the in vivo bone formation.

## Abbreviations

ALP: Alkaline phosphatase
AZR: Alizarin red
BGLAP: Osteocalcin
BLAST: Basic local alignment search tool
BM: Bone marrow
C2H2: Cis(2) His(2)
COL1A1: Collagen 1
DAVID: Database for annotation, visualization and integrated discovery
ECM: Extracellular matrix
FDR: False discovery rate
HA/TCP: Hydroxyapatite tricalcium phosphate
hMSC: Human mesenchymal stem cell
hMSC-TERT: Telomerised human mesenchymal stem cell line
KRAB: Krueppel-associated box
OB: Osteoblasts
RUNX2: Runt domain-containing transcription factor
SNP: Single nucleotide polymorphism
SOM: Self organising map
TF: Transcription factors
ZBTB38: Zinc finger- and BTB-domain containing protein 38
ZNF25: Zinc finger protein 25
ZNF608: Zinc finger protein 608
